# Insights into Food Perception and Consumer Behavior

**DOI:** 10.3390/foods13131964

**Published:** 2024-06-21

**Authors:** Wilma Maria Coelho Araújo, Sandra Fernandes Arruda

**Affiliations:** Department of Nutrition, Faculty of Health Sciences, University of Brasília, Brasília 70910-900, Brazil; sandrafarruda@gmail.com

This Special Issue contains ten articles discussing a range of topics including the role of food certification in food product choice, information highlighted on food labels, selection of “free from” products, sustainability aspects in food services, food security, and consumer understanding of the food classification proposed by the food groups in the Food-based Dietary Guidelines.

According to the *Codex alimentarius*, certification is a procedure through which official certification organizations, and those officially recognized, provide written or equivalent assurance that foods or food control systems comply with requirements. To this end, food certification is based on a series of activities that may include continuous inspection, auditing of quality systems, and product analysis. In this line of reasoning, study investigated how digital marketing can influence Romanian behavior regarding the choice of certified product, considering the association of their preferences in relation to socio-demographic characteristics and identifying consumer knowledge about the certification of agricultural and food products, as well as the reason that led them to purchase such products. The research data showed that the motivations were strongly influenced by digital marketing. The Romanian consumer was motivated by claims such as “healthier, higher quality and beneficial for the environment”, indicating the credibility of the information conveyed and showing that this channel is very important for reducing information asymmetry (contribution 1, [1,2,3]).

The unsustainable consumption and production practices significantly contribute to climate change, biodiversity loss, and environmental pollution. Methane emissions, particularly from agricultural activities associated with ruminant livestock, play a substantial role in these issues. Excessive consumption of poultry and livestock products has adverse consequences for human health and the environment, especially due to intensive and large-scale industrial farming practices that negatively impact animal welfare. Reducing the livestock population and promoting animal-friendly practices are effective strategies for mitigating greenhouse gas effects. In response to consumer preferences, companies such as McDonald’s in the UK and Carrefour Taiwan have started offering animal welfare-friendly products. Studies indicate that customers care about the well-being of farm animals and are ready to pay a premium for products that prioritize this aspect. Considering these points of view, a search employed the value–attitude–behavior (VAB) model as a theoretical basis to explore the behavioral intentions of Taiwanese consumers to buy cage-free eggs, assessing key variables such as ‘human–nature connectedness’, ‘trust in labels’, and ‘attitude toward animal welfare’. The findings showed that consumers prioritize trust in labels and animal welfare when buying cage-free eggs. The limited knowledge in Taiwan about products that promote animal welfare, and their associated labels, highlights the necessity for increasing public awareness of these items (contribution 2, [4,5]).

The sustainability of the food system is one of the main challenges facing humanity. The agri-food system needs to transition toward a more balanced system from an economic, social, and environmental point of view (contribution 3). The perceptions of the population about the existing food systems and their willingness to change their food habits are key in this transition. On the other hand, the sustainability can has about the sustainable consumption rather than sustainable production. Young people are a key demographic group to consider as they are open to new trends in consumption, including sustainable buying practices [5]. In this context, a survey was carried out with 1940 students from four universities in Spain using a specific questionnaire to identify and characterize the perceptions of young people in the university about the issue. The authors showed that a significant proportion of young people are committed to the sustainability of their food consumption habits. However, studies indicate that some young people are demanding deep and soft sustainability measures. The authors highlight that public universities have a key role in working with intrinsic and extrinsic drivers to promote food transition among young people (contribution 3, [5]).

Consumer choices have complex influences and are intricately woven into culture, society, and individual preferences, making attitudes toward many food-related decisions crucial. Culture is often a component left out of nutrition or food security plans [6]. In East Africa, diverse cultures collide with a complex tapestry of traditions. Consumer behavior theory postulates that knowing a product has specific desirable attributes or the desired attributes are inferred based on cues received from credible sources. At the same time, a revolutionary movement is emerging within the artisan industry, where artisan cooperatives are developing as social networks seeking to connect local artisans to information, resources, and markets. These cooperatives not only act as a place to provide dignified work but also provide other social benefits such as nutritional education programs (contribution 4). Considering this hypothesis, a study investigated the impact of artisan cooperatives on East African women related to changes in consumer perception and food choice. The data obtained indicated that when given a choice, women did not always eat healthier despite having more money and information about healthy diets. Similarly, the authors revealed that culture and location impacted how these women cooked, what food was available to them, and what foods they chose to consume. The women also explained how a higher income provided greater agency when buying food (contribution 4).

In the study titled ‘How Do Brazilian Consumers Understand Food Groups in the Food-based Dietary Guidelines?’ (contribution 5), the authors aimed to evaluate how a sample of 902 individuals understood the food group classifications in the Food-based Dietary Guidelines (FGDGs). The data indicated that consumers believe it is easier to classify foods according to food groups. However, although consumers traditionally can easily recognize foods according to their origin—animal or vegetable—the authors still identify asymmetries regarding the inclusion of food items in the group of species from the animal kingdom and in the group of species from the plant kingdom. They concluded that this exploratory study highlights important information that can contribute to improving the FGDGs. It is essential to consider the consumer’s understanding and guide them regarding choices from a technical point of view. Food is an important source of nutrients for maintaining our vital activities; the balance between the quantity and quality of nutrients must be geared toward achieving the best quality of health [7].

Today, mindful selection of foods, following a particular diet, and healthy and sustainable eating habits continue to be more and more important for consumers (contribution 6). In this sense, nutritional labeling is an important vehicle to inform the consumer about the properties of the food in terms of nutritional value and nutritional claims, which include any statement indicating that a food has positive nutritional properties related to its energy value or nutrients content, including claims of absolute, comparative, and no added content [8]. The popularity of “free-from” food products, which exclude several ingredients such as lactose, gluten, or sugar, is increasing globally. However, experts agree that avoiding these ingredients without medical reasons can lead to nutritional deficiencies. The motivation behind these choices is usually supported by several factors: it may originate from health issues, food allergies or intolerances, risk-avoiding behavior, or even due to sustainability aspects for which the consumer may choose to avoid certain ingredients. At the same time, subconscious heuristics and situational cues also influence consumers’ decision-making process about food purchases, which makes uncovering motivations challenging [9,10].

A study evaluated the extent of unnecessary “free-from” consumption (regular consumption without medical justification), with a focus on gluten- and lactose-free food products and called attention to the problem of the misperception of this product category. The study’s results show that the “free-from customers” who most frequently consume both lactose- and gluten-free foods are mostly women. Because they are responsible for their choices and the food choices of their family members, they are willing to pay more for these products, even without being sure of the need to consume them. In general, choices are based on self-diagnosis and the belief that such products are healthier compared to the originals. The authors highlight the lack of information among Hungarian consumers and in general for European consumers, emphasizing the importance of monitoring health, through medical examinations, to minimize risks of nutritional deficiencies in the medium and long term and guarantee the purchase of products best suited to healthy living (contribution 6).

Following this logic, another study evaluated the effects of teosinte flour obtained from seeds on selected physicochemical characteristics and consumer perceptions of gluten-free cocoa cookies formulated with mung bean (*Vigna radiata*) flour. The levels of teosinte flour did not significantly affect the acceptability of appearance, color, texture, flavor, aroma, and overall quality, nor did they effect the responses relating to overall liking and purchase intent. The authors concluded by highlighting the importance of using new food sources that allow customers to obtain products with an excellent nutritional profile and that provide health benefits (contribution 7).

Eating is a voluntary and conscious process of choosing from the food substances available, as well as preparation, ingestion, and absorption. Nutrition refers to the transformation of food substances into simpler substances (nutrients) that will be absorbed by the body. A meal is part of the daily diet and presupposes the intake of nutrients in quantity, quality, harmony, proportionality, and adequacy. On the other hand, the socializing effects of eating in groups impact the harmonization and strengthening of emotional bonds, thus stabilizing social structures [11]. Therefore, the choice of menu items can be emotional, instinctive, irrational, and without consideration of information about the sugar content, the fat content, and what type of damage it could cause. The advent of the digital era can infer daily food choices in food service, for example, the use of QR codes for food ordering in restaurants or on platforms such as Uber Eats (contribution 8).

As part of the so-called nudging approach, the use of signals such as color can be useful in influencing consumer decision making. In this sense, research was carried out with the aim of investigating whether the position of menu items influences consumer choice, how this choice would be made, and considering the availability of general information, whether this would have any association with the item’s position. And, finally, the authors investigated whether price affected choices in the relationship between position and recommendation. The data obtained are in accordance with self-reported facts and that the general recommendations did not influence the consumer as could have been expected, contrary to the literature, possibly due to the limitation of blatant marketing tactics and the fact that more studies are needed to obtain data accurate (contribution 8).

Food neophobia refers to customers being fearful of trying new foods. This behavior is characterized by their refusal of unfamiliar foods. Heredity, hypersensitivity to bitter taste, restrictive eating practices, cultural and socio-demographic characteristics, and negative emotions associated with exposure to food are some of the factors that can contribute to food refusals in childhood, adolescence, and adulthood. In this way, personal and family knowledge and experience influence people’s interest in trying foods that are different from their usual choices. Considering that globalization can contribute to the dissemination of food culture among people, it is crucial to identify the difference between food neophobia and food preferences among consumers [12].

The Food Neophobia Scale (FNS) is an instrument which evaluates individuals’ hesitancy or resistance toward trying new or unfamiliar foods. The FNS was originally developed in English and has been translated into Swedish, Finnish, Spanish, European Portuguese, Brazilian Portuguese, German, Hungarian, and French. To assess food neophobia in the Romanian population, the FNS questionnaire was translated into Romanian, performed its cultural adaptation, assessing its validity using a national study group while also comparing the results with those from various other countries (contribution 9). The data obtained among Romanian consumers show a spectrum of attitudes toward novel foods, with a notable prevalence of neutrality and a substantial number displaying an aversion or reluctance toward unfamiliar food items. The adoption of a validated national scale to investigate food neophobia contributes to identifying globally prevalent eating behaviors and strengthens its universal applicability, facilitating its utilization in multicentric studies across various countries. The data obtained highlighted the coexistence of both a reluctance toward unfamiliarity and an eagerness to explore novelties among the respondents. The identification of neophilic and neophobic tendencies in the researched population shows the complexity in interpreting consumer food preferences and attitudes toward culinary exploration. At the same time, it helps researchers, healthcare professionals, the food industry, public health organizations, and consumers to disseminate insights into dietary behaviors, favoring the development of intervention strategies, products, and public health initiatives that aim to promote diverse eating habits and nutritious (contribution 9, [12]).

Over 200 diseases are caused by eating food contaminated with bacteria, viruses, parasites, or chemical substances such as heavy metals. This growing public health problem is having a considerable socioeconomic impact though strains on healthcare systems, lost productivity, and harm caused to tourism and trade. These diseases contribute significantly to the global burden of disease and mortality [13].

Foodborne diseases are caused by the contamination of food and occur at any stage of the food production, delivery, and consumption chain. They can result from several forms of environmental contamination including pollution in water, soil, and air, as well as unsafe food storage and processing. Individual consumers and food handlers play a huge role in preventing foodborne diseases [13].

Consumers’ food safety knowledge and their perceptions of food-associated risks can influence their food handling practices in the home. A risk perception is an individual or group judgment of the magnitude and likelihood of a negative outcome from an action. In general, consumers perceive low-moisture foods, such as tree nuts, as presenting a low risk for foodborne illnesses and believe that tree nuts have many health benefits [14,15].

The consumption of tree nuts and products made from them has increased in recent years, in part due to their purported nutritional value and health benefits. As a low-moisture food (water activity (aw) of <0.85), tree nuts do not provide sufficient moisture to support pathogen growth, but some foodborne pathogens such as Salmonella have developed the ability to survive at lower water activities for long periods of time. Models for understanding behavior changes have been used to explain the relationships between knowledge, perceptions, and behavior. To investigate the gap data regarding the risks and benefits of tree nut handling to promote safe handling practices and prevent foodborne illnesses, research was carried out based on planned behavior (TPB) of the consumer. The theory of planned behavior (TPB) predicts the intention of individuals to perform a given behavior based on their attitudes, subjective norms, and perceived behavioral control, thus being able to explain food safety behavior via risk perceptions (contribution 10). 

The results showed that consumers with a lower income had lower levels of food safety knowledge and risk perception than high-income consumers, were less likely to purchase tree nuts, and may be less concerned about tree nut food safety. The consumers who held an associate degree or lower had lower levels of food safety knowledge and risk perception. Those with less education have been reported to have less knowledge of food safety and to lack safe handling behaviors, which aligns with the present study. Consumers living in metropolitan areas have been reported to engage in behavior that is more risk than that of nonmetropolitan consumers. Such data determine the importance of consumers understanding food safety, its implications, and the factors that influence their behavior when handling food, in order to minimize the occurrence of food-borne illnesses (contribution 10).

**Conclusions**

Scientific knowledge about food science has facilitated the development of technologies to preserve, conserve, process, and modify different types of foods identified worldwide, as well as ensuring the supply of safe foods. However, the consumer did not follow scientific advances, and concerns about food safety intensified in parallel with the growth in production and the development of new technologies. The studies described in this Special Issue point to the need for new research that also ratifies the nutritional quality of industrialized products, the importance of different types of certifications as a quality requirement, the importance that we, the consumers, must perform sustainable consumption of food, and how vital it is that inputs used in the production chain can demystify the supposed belief that industrialized products are not safe.

The informational asymmetry between producer, consumer, and regulatory bodies is one of the factors that contributes to consumer disbelief regarding the safety and reliability of the product and the manufacturer, even with advances in the labeling of industrialized products that identify, in addition to the name of the product, the list of ingredients, nutritional information, and property claims on the labels, thus rendering the understanding of this information often restricted to professionals in the field.

Therefore, it is recommended that research into consumer perception, knowledge and understanding of food production and industrialization, and other aspects relating to this topic be carried out. While quantitative research assumes “nanometric dimensions” that allow a great depth of understanding about the composition and technology for new foods, consumers are still unaware of the classification of foods according to food groups.
